# Enfortumab vedotin plus pembrolizumab in treatment‐naïve metastatic urothelial carcinoma patients: An Austrian real‐world analysis

**DOI:** 10.1002/ijc.70203

**Published:** 2025-10-22

**Authors:** Dora Niedersuess‐Beke, Karl Mayrhofer, Johanna Krauter, Johannes Franke, Dominic Vais, Maximillian Pallauf, David Kiesl, Ferdinand Luger, Jacob Pfuner, Angelika Terbuch, Thomas Bauernhofer, Jasmin Spielgelberg, Andreas Banner, Stefan Aufderklamm, Clemens Wiesinger, Susanne Schnabel, Simon Peter Gampenrieder, Josef Mühlmann, Sonia Vallet, Sabine Weibrecht, Franz Stoiber, Haleh Andalibi, Harun Fajkovic, Hossein Taghizadeh, Jan Miechowiecki, Roman Taedcke, Daniel Heintel, Shahrokh F. Shariat, Martin Pichler, Wolfgang Hilbe, Renate Pichler

**Affiliations:** ^1^ Department of Internal Medicine I., Centre for Oncology and Haematology Vienna Healthcare Group Ottakring Vienna Austria; ^2^ Department of Urology Medical University of Vienna Vienna Austria; ^3^ Department of Internal Medicine Oncology and Haematology State Hospital Klagenfurt Klagenfurt Austria; ^4^ Department of Urology, University Hospital Salzburg Paracelsus Medical University Salzburg Salzburg Austria; ^5^ Department of Internal Medicine I with Haematology, Medical Oncology, Ordensklinikum Linz Elisabethinen, Linz Austria and Johannes Kepler University Linz Austria; ^6^ Department of Urology Ordensklinikum Linz Elisabethinen Linz Austria; ^7^ Department of Urology Klinikum Donaustadt Vienna Austria; ^8^ Division of Clinical Oncology, Department of Internal Medicine Medical University Graz Graz Austria; ^9^ Department of Oncology, Hematology and Palliative Medicine Klinikum Oberwart Oberwart Austria; ^10^ Department of Urology Vienna Healthcare Group, Favoriten Vienna Austria; ^11^ Department of Urology State Hospital Bregenz Bregenz Austria; ^12^ Department of Urology Klinikum Wels‐Grieskirchen Wels Austria; ^13^ Department of Internal Medicine III with Haematology, Medical Oncology, Haemostaseology, Infectiology and Rheumatology, Oncologic Centre Paracelsus Medical University Salzburg Salzburg Austria; ^14^ Department of Internal Medicine St. John of God Hospital Salzburg Austria; ^15^ Department of Internal Medicine II University Hospital Krems and Karl Landsteiner University of Health Sciences Krems an der Donau Austria; ^16^ Department of Internal Medicine II St. John of God Hospital Vienna Austria; ^17^ Department of Urology State Hospital Salzkammergut Vöcklabruck Austria; ^18^ Department of Internal Medicine Oncology and Haematology Barmherzige Schwestern Vienna Austria; ^19^ Department of Urology and Andrology University Hospital St. Pölten St. Pölten Austria; ^20^ Department for Urology Karl Landsteiner Institute for Urological Research and Training St. Pölten Austria; ^21^ Department of Oncology, University Hospital St. Pölten St. Pölten Austria; ^22^ Department of Haematology and Oncology Hanusch Krankenhaus of the OEGK, Hanusch Hospital Vienna Austria; ^23^ Department for Oncology Karl Landsteiner Institute for Bioanalytical Oncology Horn Austria; ^24^ Department of Internal Medicine Oncology and Haematology Klinik Oberwart Oberwart Austria; ^25^ Department of Urology, Comprehensive Cancer Centre Innsbruck Medical University of Innsbruck Innsbruck Austria

**Keywords:** enfortumab vedotin, metastatic urothelial carcinoma, pembrolizumab, real‐world evidence, registry

## Abstract

Antibody‐drug conjugates (ADCs) and immunotherapy have changed the treatment landscape for locally advanced and metastatic urothelial carcinoma (la/mUC). Clinical trials have demonstrated the superiority of enfortumab vedotin (EV) combined with pembrolizumab (P) over chemotherapy. This retrospective, multicentre Austrian registry analysis evaluated the effectiveness and safety of EV + P in treatment‐naïve la/mUC patients in a real‐world setting. This study included 203 first‐line EV + P treated patients from 20 Austrian centres. The majority were male (77.3%), with a median age of 70 years (range, 26–92), while 33.5% were ≥75 years. Comorbidities were common, with 25.1% having a Charlson Comorbidity Index (CCI) ≥ 5, and 46.3% moderate to severe renal impairment. Among 195 evaluable patients, the objective response rate (ORR) was 63.6% (95% CI, 56.6–70.0), including 21.5% complete responses and 42.1% partial responses. Disease control was achieved in 75.4% (95% CI, 68.9–80.9). Median progression‐free survival (PFS) and overall survival (OS) were not reached after a median follow‐up of 5.8 months. Poor outcomes were associated with ECOG PS >2 (PFS, *p* = .003; OS, *p*< .001) and CCI ≥5 (PFS, *p* = .011; OS, *p* = .08). EV + P was generally well tolerated; grade ≥3 treatment‐related adverse events occurred in 38.4% of patients and immune‐related adverse events in 19.2%. Limitations of this study include its retrospective design and short follow‐up. These findings support the clinical use of EV + P as first‐line treatment for la/mUC, demonstrating substantial effectiveness and manageable toxicity in a large real‐world population, including elderly and comorbid patients. Extended follow‐up is warranted to determine long‐term survival and predictive markers of response.

## Graphical abstract

What's new?

New antibody‐drug conjugates (ADCs) and immunotherapies have helped expand treatment options for urothelial carcinoma (UC). Clinical trials suggest that the ADC enfortumab vedotin (EV) and the immunotherapeutic agent pembrolizumab, when used in combination, are especially effective against UC. In this study, the authors examined the effectiveness of EV plus pembrolizumab in routine clinical practice in patients with treatment‐naïve locally advanced or metastatic UC (la/mUC). Analyses reveal high treatment response rates, with favourable responses even among those with comorbidities such as impaired renal function. The findings highlight the clinical promise of EV plus pembrolizumab as a first‐line treatment for advanced UC.

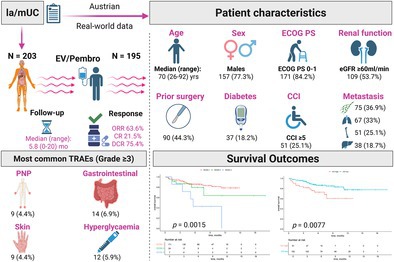

**Abbreviations**
ADCs: antibody‐drug conjugates
AE: adverse event
AESI: adverse event of special interest
BMI: body mass index
CCI: Charlson comorbidity index
CI: confidence interval
CKD: chronic kidney disease
CPS: Combined Positive Score
CR: complete response
CT: computed tomography
CTCAE: Common Terminology Criteria for Adverse Events
DCR: disease control rate
ECOG PS: Eastern Cooperative Oncology Group Performance Status
eGFR: estimated glomerular filtration rate
EMA: European Medicines Agency
ESMO: European Society for Medical Oncology
EV: enfortumab vedotin
FDA: Food and Drug Administration
FGFR: fibroblast growth factor receptor
GFR: glomerular filtration rate
ICI: immune checkpoint inhibitor
IrAE: immune‐related adverse event
la: locally advanced
m: median
MMAE: monomethyl auristatin E
MRI: magnetic resonance imaging
mUC: metastatic urothelial carcinoma
NA: not available
NR: not reached
NU: nephroureterectomy
ORR: objective response rate
OS: overall survival
P: pembrolizumab
PD‐L1: Programmed Death Ligand 1
PFS: progression‐free survival
PR: partial response
RC: radical cystectomy
SAE: severe adverse event
SD: stable disease
TRAE: treatment‐relative adverse event
UC: urothelial carcinoma

## 1 INTRODUCTION

Urothelial carcinoma (UC) is the most prevalent malignancy of the bladder and urinary tract, with over 613,000 new cases of bladder cancer diagnosed in 2022 and a mortality rate of 36%.^1^, ^2^ Locally advanced or metastatic UC (la/mUC) is associated with poor prognosis, with survival outcomes influenced by both the treatment approach and patient‐specific factors.^3^

The treatment landscape for advanced UC has evolved significantly with the introduction of antibody‐drug conjugates (ADCs) and immunotherapy.^4^ Enfortumab vedotin (EV) is a highly potent ADC designed to target nectin‐4, a cell adhesion molecule that is highly expressed in UC cells. The therapeutic mechanism of EV involves a nectin‐4‐specific monoclonal antibody conjugated with the microtubule‐disrupting agent monomethyl auristatin E (MMAE), which induces cell‐cycle arrest and apoptosis in the targeted cells. EV was initially approved as monotherapy for patients who had progressed after receiving standard‐of‐care treatment.^5^, ^6^

Until recently, the first‐line standard of care for patients with advanced UC included platinum‐based chemotherapy and immune checkpoint inhibitors (ICIs). In the phase 3, global, open‐label, randomised EV‐302 trial (NCT04223856), EV in combination with pembrolizumab (P), a PD‐1 inhibitor, demonstrated superior efficacy over platinum‐based chemotherapy in patients with previously untreated locally advanced or metastatic UC (la/mUC).^7^ Based on these positive outcomes, the combination of EV plus P received approval from the US Food and Drug Administration (FDA) in December 2023 and from the European Medicines Agency (EMA) in August 2024,^7^, ^8^, ^9^ establishing it as the first‐line standard therapy.^7^ Consequently, the European Society for Medical Oncology (ESMO) clinical practice guideline was recently updated to recommend EV plus P as the new first‐line standard‐of‐care therapy for UC.^10^

A recent publication presented Austrian real‐world evidence on EV monotherapy in previously treated patients with mUC.^11^ This study introduces a new retrospective analysis from an EV‐treated patient registry in Austria, aiming to evaluate patient characteristics, treatment duration, effectiveness, and safety of the EV plus P combination in a real‐world setting.

## 2 PATIENTS AND METHODS

### 2.1 Analysed population

A retrospective, non‐interventional, multicentre analysis was performed based on registry data of patients with locally advanced/metastatic UC (la/mUC) treated as first‐line therapy with EV plus P. The registry included any patient who had received at least one dose of EV plus P during the analysed period. The objective of this analysis was to achieve a more comprehensive understanding of the duration of treatment, its effectiveness (objective response rate [ORR], disease control rate [DCR], progression‐free survival [PFS], overall survival [OS]), and its safety.

### 2.2 Data collection

A retrospective data collection was conducted from multiple Austrian oncology and urology centres in which patients had initiated EV + P between September 2023 and December 2024, with a follow‐up period until the beginning of March 2025. This collection was initiated as an early access programme in Austria prior to the EMA approval of EV plus P in August 2024. The data collected by the treating physicians included patient demographics and clinical characteristics, treatment response, and safety information; following anonymisation, the data was transferred for statistical analysis.

### 2.3 Assessments

After the start of treatment with EV plus P, computed tomography (CT) and/or magnetic resonance imaging was used for assessing treatment response over time. Treating physicians retrospectively assessed tumour responses in accordance with RECIST v1.1 criteria; those RECIST v1.1 assessments were only investigator‐assessed.

The Common Terminology Criteria for Adverse Events (CTCAE) version 5.0 was used to classify the adverse events (AEs), including some AEs of special interest (AESIs).

### 2.4 Statistical analysis

Patient characteristics and the ORR were described using summary statistics. The corresponding 95% confidence intervals (CIs) were subsequently derived for PFS and OS, which were analysed through the Kaplan–Meier method. Patients without documented progression (PFS) or who were lost to follow‐up (OS) or who were still alive at the time of the data cut‐off date were censored at that time or the last contact. Missing data were not imputed. Datatab.net, “R” statistical computing (Kaplan–Meier curves) and socscistatistics.com (Chi^2^ analyses) were used to conduct all statistical analyses, with *p*‐values calculated using the Log‐Rank test. A two‐sided *p*‐value <.05 was considered to be statistically significant.

## 3 RESULTS

### 3.1 Patient and disease characteristics

A total of 203 patients with la/mUC were treated with EV plus P in 20 Austrian oncology and urology centres. The baseline patient demographics and disease characteristics are presented in Table 1. Most patients (*n* = 157, 77.3%) were male; the median age was 70 years (range, 26–92), and 33.5% (*n* = 68) were aged ≥75 years. At the time of treatment initiation, most patients had a good performance status (ECOG PS 0–1: *n* = 171, 84.2%), while 11.8% (*n* = 24) were classified as ECOG PS 2 and 3.4% (*n* = 7) as ECOG PS 3.

**TABLE 1:** Demographics, clinical, and disease characteristics of patients (*N* = 203).

Demographics and clinical characteristics	*n* (%)^a^, ^b^
Age	
Median age, years (range)	70 (26–92)
<75	135 (66.5)
≥75	68 (33.5)
Gender	
Female	46 (22.7)
Male	157 (77.3)
ECOG performance status	
0	107 (52.7)
1	64 (31.5)
2	24 (11.8)
3	7 (3.4)
NA	1 (<1)
BMI, kg	
Median (range)	24.9 (13.3–37.9)
<30	159 (78.3)
≥30	21 (10.3)
NA	23 (11.3)
Charlson comorbidity index	
Median score	3
Low (score 0–4)	152 (74.9)
High (score ≥5)	51 (25.1)
Diabetes mellitus	
Yes	37 (18.2)
No	166 (81.8)
Renal function	
No renal insufficiency to mild CKD (eGFR ≥60 mL/min)	109 (53.7)
Moderate CKD (eGFR 30–59 mL/min)	85 (41.9)
Severe CKD (eGFR <30 mL/min)	9 (4.4)

Disease characteristics, *n* (%)	
*Location of metastases*	
Lymph nodes	75 (36.9)
Lungs	67 (33.0)
Liver	38 (18.7)
Bone	51 (25.1)
Brain	2 (1.0)
Others^c^	33 (16.3)
*PD‐L1 status*	
CPS ≥10	58 (28.6)
CPS <10	68 (33.5)
NA	77 (37.9)
*FGFR 3 alterations*	
Positive	16 (7.9)
Negative	71 (35.0)
NA	116 (57.1)
*Histology subtype of primary disease*	
Urothelial carcinoma (*UC*)	176 (86.7)
Mixed type	11 (5.4)
Squamous cell carcinoma	3 (1.5)
Sarcomatoid differentiation	3 (1.5)
Plasmazytoid differentiation	3 (1.5)
Others^d^	7 (3.4)

Treatment history, *n* (%)	
*Prior definitive therapy of primary tumour*	
RC/NU	90 (44.3)
Radiochemotherapy	7 (3.4)
None	106 (52.2)
(*Neo*) *Adjuvant therapy*	
Received	63 (31.0)
None	140 (69.0)

Abbreviations: BMI, body mass index; CKD, chronic kidney disease; CPS, combined positive score; ECOG, Eastern Cooperative Oncology Group; eGFR, estimated glomerular filtration rate; FGFR, fibroblast growth factor receptor; GFR, glomerular filtration rate; NA, not available; NU, nephroureterectomy; PD‐L1, programmed death‐ligand 1; RC, radical cystectomy; UC, urothelial cancer.

^a^ Percentages may not equal 100 because of rounding.

^b^ Although other indicated.

^c^ Inclusive local recurrence (*n* = 13), peritoneum (*n* = 7), muscular (*n* = 4), adrenal gland (*n* = 3), pleura (*n* = 3), cutaneous (*n* = 2), pancreas (*n* = 2), splenum (*n* = 2), and pericard (*n* = 1).

^d^ Inclusive basal cell type, adenosquamous, micropapillary, small cell carcinoma, trophoblastic subtype, nested or glandular histology.

Most patients (*n* = 152, 74.9%) had a low Charlson comorbidity index (CCI); however, 94 patients (46.3%) suffered from moderate to severe renal insufficiency. Metastases were predominantly located in the lymph nodes (*n* = 75, 36.9%), lungs (*n* = 67, 33.0%), and liver (*n* = 38, 18.7%). Overall, 28.6% (*n* = 58) of patients had a tumour with a high PD‐L1 expression measured with the Combined Positive Score (CPS ≥10). Most patients (*n* = 176, 86.7%) had pure UC histology, and 44.3% (*n* = 90) had undergone definitive local therapy for the primary tumour (radical cystectomy or a nephroureterectomy) before initiating systemic treatment.

### 3.2 Effectiveness

The median treatment duration was 4.7 months (range, 0.7–17.4) for EV and 4.5 months (range, 0.7–20.7) for P (Table 2A). Among evaluable patients (*n* = 195), the ORR was 63.6% (95% CI, 56.6–70.0), with 42 patients (21.5%) achieving a radiologic complete response (CR) and 82 patients (42.1%) a partial response (PR). The DCR rate reached 75.4% (95% CI, 68.9–80.9), with 11.8% of patients with stable disease (SD).

**TABLE 2:** Effectiveness of enfortumab vedotin (EV) plus pembrolizumab (P) in evaluable patients with locally advanced/metastatic urothelial carcinoma (Ia/mUC) (*N* = 195).

(A) Treatment response in overall analysed population	
Treatment response	*n* (%)^a^
Duration of treatment	
Median, months (range)	
EV	4.7 (0.7–17.4)
P	4.5 (0.7–20.7)
Number of therapy cycles^b^ administered	
Median, *n* (range)	
EV	12 (1–39)
P	6 (1–26)
Objective response rate (ORR), % (95% CI)	63.6 (56.6–70.0)
Disease control rate (DCR), % (95% CI)	75.4 (68.9–80.9)
Best response, *n* (%)	
Complete response (CR)	42 (21.5)
Partial response (PR)	82 (42.1)
Stable disease (SD)	23 (11.8)
Progressive disease (PD)	48 (24.6)
Progression‐free survival (PFS)	
Median follow‐up, months	5.8
Median, months (95% CI)	NR (11.7‐NR)
6‐month PFS, % (95% CI)	72.3 (65.9–79.4)
12‐month PFS, % (95% CI)	59.6 (50.3–70.7)
Overall survival (OS)	
Median follow‐up, months	5.8
Median, months (95% CI)	NR (NR–NR)
6‐month OS, % (95% CI)	83.2 (77.7–89.1)
12‐month OS, % (95% CI)	73.6 (65.0–83.4)

(B) Subgroup analyses of objective response rate (ORR)								
Effectiveness parameter	Overall (*N* = 195)	Patient specific characteristics						
		Renal function		Charlson comorbidity index (CCI)		ECOG PS		
		eGFR ≥30 (*n* = 186)	eGFR <30 (*n* = 9)	Low (0–4) (*n* = 146)	High (≥5) (*n* = 49)	0–1 (*n* = 164)	2 (*n* = 23)	3 (*n* = 7)
Objective response rate (ORR), % (95% CI)	63.6 (56.6–70.0)	64.5 (57.4–71.0)	44.4 (18.8–73.4)	67.1 (59.1–74.2)	53.1 (39.4–66.3)	64.0 (54.6–71.7)	69.6 (48.9–84.6)	28.6 (7.6–64.8)
*p*‐value	‐	.22		.08		.058^c^	.053^d^	

Effectiveness parameter	Overall (*N* = 195)	Patient specific characteristics					
		Liver metastases		Bone metastases		Platinum fitness	
		Present (*n* = 36)	Absent (*n* = 159)	Present (*n* = 49)	Absent (*n* = 146)	Fit (*n* = 126)	Unfit (*n* = 69)
Objective response rate (ORR), % (95% CI)	63.6 (56.6–70.0)	52.8 (37.0–68.0)	66.0 (58.4–73.0)	42.9 (30.0–56.7)	70.5 (62.7–77.4)	64.3 (55.6–72.1)	62.3 (50.5–72.8)
*p*‐value	‐	.14		<.001^e^		.78	

Abbreviations: CI, confidentiality interval; EV, enfortumab vedotin; NA, not available.

^a^ Although other indicated.

^b^ 1 cycle = 2 administrations of EV or 1 administration of pembrolizumab.

^c^ ECOG PS 0–1 versus ECOG PS 3.

^d^ ECOG PS 2–3 versus ECOG PS 2.

^e^ Statistically significant value.

**FIGURE 1:**
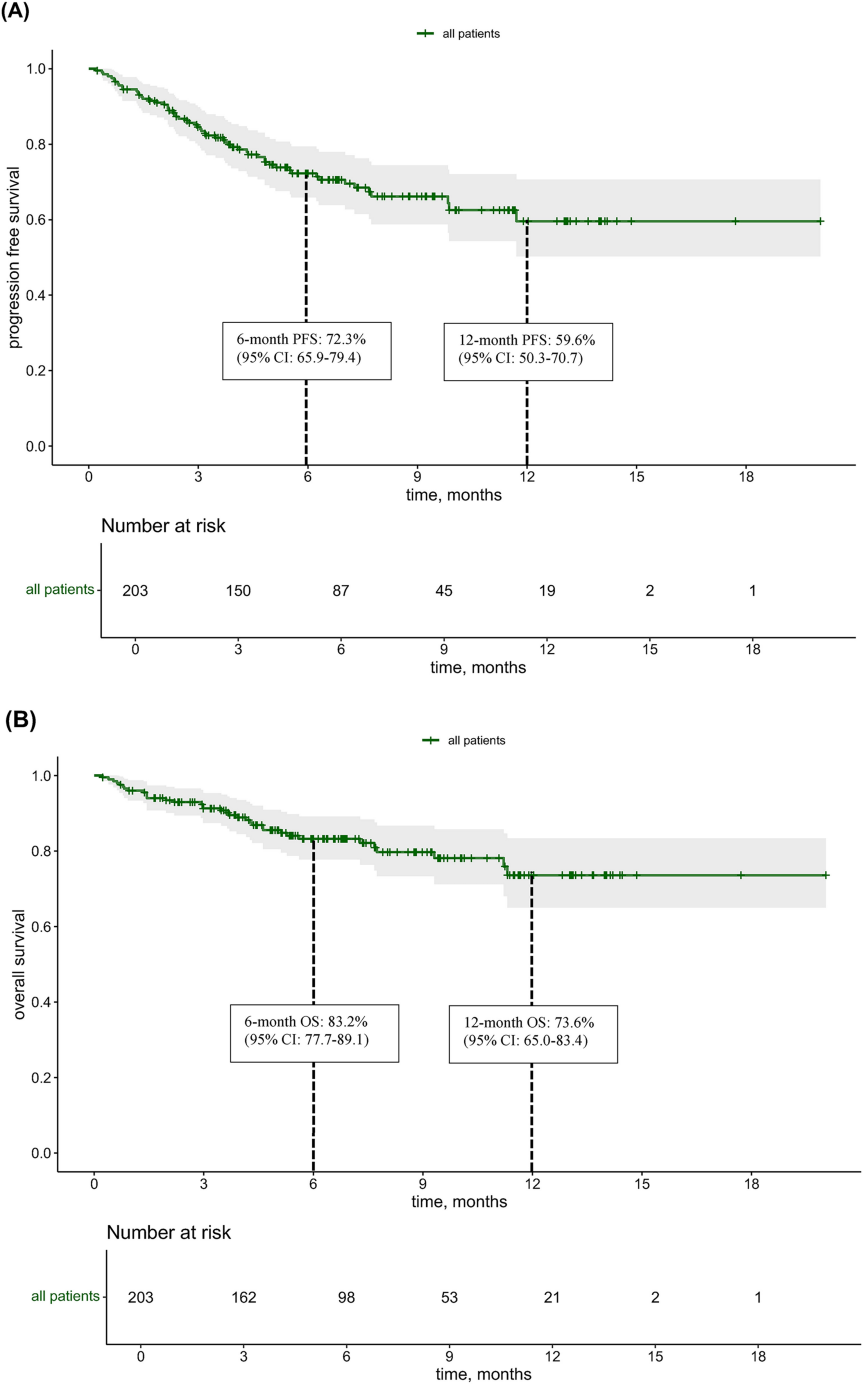
Kaplan–Meier plots of median progression‐free survival (PFS) (A) and median overall survival (OS) (B) (*N* = 203).

After a median follow‐up of 5.8 months (range 0.2–20), the median (m) PFS (95% CI, 11.7‐NR) and mOS (95% CI, NR‐NR) had not been reached (Figure 1). The 6‐month PFS was 72.3% (95% CI: 65.9–79.4) and the 12‐month PFS 59.6% (95% CI: 50.3–70.7), while the 6‐month OS was 83.2% (95% CI: 77.7–89.1) and the 12‐month OS 73.6% (95% CI: 65.0–83.4). At the data cut‐off date (2 March 2025), 35 patients (17.2%) had died. Therapy was still ongoing in 96 patients (47.3%) receiving EV and 114 patients (56.2%) undergoing treatment with P.

**FIGURE 2:**
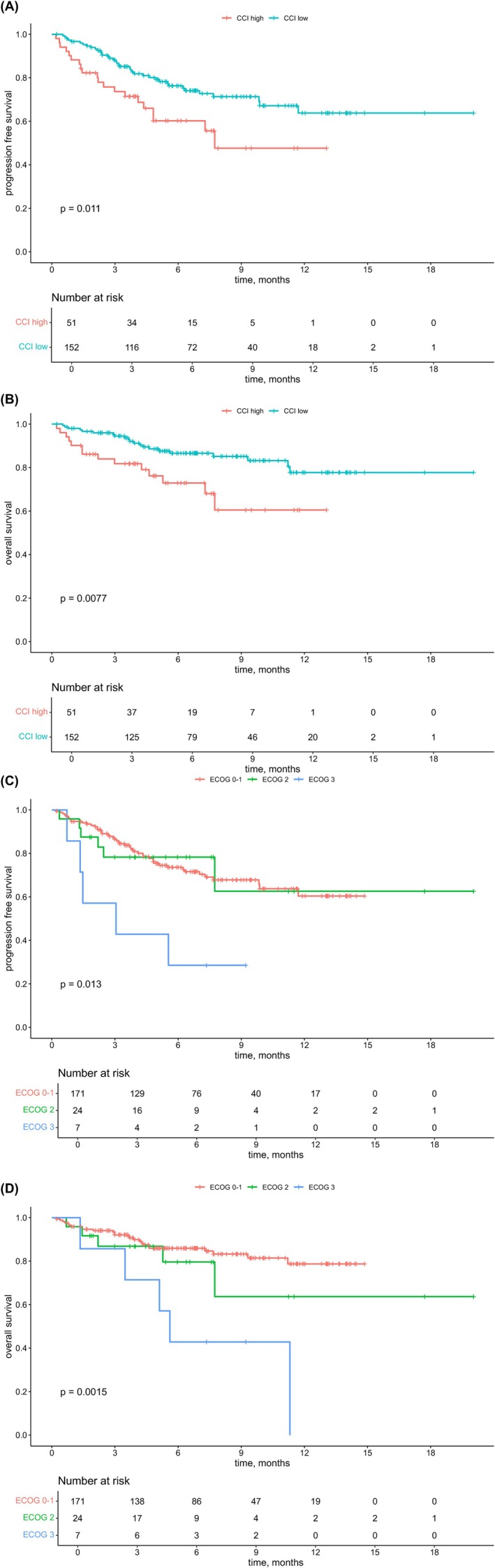
Kaplan–Meier plots of median progression‐free survival (PFS) (A) and median overall survival (OS) (B) according to Charlson comorbidity index (CCI), as well as ECOG status (PFS, C and OS, D) at treatment initiation (*N* = 203).

Focusing on subgroups, patients with a high CCI (score ≥5) had a poorer ORR compared to those with a low CCI (67.1% [95% CI, 59.1–74.2] vs. 53.1% [95% CI, 39.4–66.3]; *p* = 0.08) (Table 2B). Correspondingly, patients with a high CCI at treatment initiation had significantly shorter outcomes in terms of mPFS (7.7 months vs. NR; 95% CI, 4.8–NR; *p* = .011) (Figure 2A) and mOS (NR vs. NR; 95% CI, 7.7–NR; *p* = .008) (Figure 2B). Neither an impaired kidney function (eGFR <60) (*p* = .22), nor the histology of the primary tumour (*p* = .79) or the presence of severe adverse events (SAEs) (*p* = .99) influenced treatment response.

In contrast, an ECOG status of 3 was identified as a negative prognostic marker compared to ECOG PS 0‐2. Specifically, the ORR was 64.0% (95% CI, 54.6–71.7) for ECOG PS 0–1, 69.6% (95% CI, 48.9–84.6) for ECOG PS 2, and only 28.6% (95% CI, 7.6–64.8) for ECOG PS 3 (Table 2B). Similarly, patients with poorer PS experienced a significant reduction in PFS (0–1 vs. 3, mPFS NR vs. 3.1 months; 95% CI, 1.3–5.5; *p* = .013) (Figure 2C) and OS (0–1 vs. 3, mOS NR vs. 5.6 months; 95% CI, 3.5–5.6; *p* = 0.0015) (Figure 2D), although mPFS and mOS had not yet been reached.

Moreover, patients with bone metastases at diagnosis had a significantly poorer response compared to those without (ORR, 42.9% vs. 70.5%; *p* <.001), with a mPFS of 11.7 months (95% CI: 6.2‐NR) versus not yet reached (*p* = .177), respectively. However, this was not the case for patients with liver lesions at diagnosis (ORR, 52.8% vs. 66.0%; *p* = .14). At the data‐cut‐off date, the mPFS (*p* = .318) and mOS (*p* = .194) had not been reached for patients with or without bone metastases at diagnosis. Furthermore, the platinum fitness had no impact on the treatment response (ORR, 64.3% for fit patients vs. 62.3% for unfit patients; *p* = .78) (Table 2B).

## 4 SAFETY

Treatment‐related adverse events (TRAEs) grade 1–2 were reported in 72.9% of patients, with grade ≥3 toxicities occurring in 38.4% of patients (Table 3). The most prevalent TRAEs of grade 1–2 were peripheral sensory neuropathy (in 70 patients, 34.5%), skin disorder (*n* = 67, 33.0%), and gastrointestinal disorders (*n* = 48, 23.6%). The most frequently observed grade ≥3 TRAEs were gastrointestinal disorders (*n* = 14, 6.9%) and endocrine disorders (*n* = 12, 5.9%).

**TABLE 3:** Safety outcome (*N* = 203).

(A) Treatment‐related adverse events (TRAEs)		
TRAEs, *n* (%)^a^	Grade 1–2	Grade ≥3
Any Event	148 (72.9)	78 (38.4)
Peripheral sensory neuropathy	70 (34.5)	9 (4.4)
Skin disorder	67 (33.0)	9 (4.4)
Gastrointestinal disorder	48 (23.6)	14 (6.9)^b^
Dysgeusia/decreased appetite	22 (10.8)	‐
Eye disorder	17 (8.4)	2 (1.0)
Endocrine disorder (hyperglycaemia)	13 (6.4)	12 (5.9)^c^
Fatigue	13 (6.4)	‐
Transaminases	6 (3.0)	1 (<1)
Blood count decrease	3 (1.5)	5 (2.5)
Infection	3 (1.5)	5 (2.5)^d^
Respiratory disorder	2 (1.0)	3 (1.5)
Kidney injury	2 (1.0)	2 (1.0)
Bleeding	‐	1 (<1)^e^
Cardiovascular disorder	‐	3 (1.5)
Others	7 (3.4)	‐

(B) Immune‐related adverse events (IrAEs)		
IrAEs *n* (%)^f^	Grade 1–2	Grade ≥3
Any event	61 (30.0)	39 (19.2)
Thyroiditis	8 (3.9)	‐
Skin disorder	5 (2.5)	5 (2.5)^g^
Hepatitis	4 (2.0)	8 (3.9)
Pneumonitis	3 (1.5)	6 (3.0)
Endocrine disorder	2 (1.0)	7 (3.4)
Colitis	1 (<1)	7 (3.4)
Arthritis	1 (<1)	‐
Nephritis	‐	1 (<1)
Pancreatitis	‐	1 (<1)

*Note*: TRAEs, treatment‐relative adverse events, which were graded as per Common Terminology Criteria for Adverse Events (CTCAE) version 5.0, as determined by the treating physician.

^a^ Percentage may not equal 100 because of rounding.

^b^ Including grade 5 enterocolitis (*n* = 1).

^c^ Including grade 5 diabetic ketoacidosis (*n* = 1).

^d^ Including grade 5 *Pneumocystis carinii* (*n* = 1), infectious disease with multi‐organ failure (*n* = 1) and sepsis (*n* = 1).

^e^ Including grade 5 rectal bleeding (*n* = 1).

^f^ Percentage may not equal 100 because of rounding; TRAEs, treatment‐relative adverse events, which were graded as per Common Terminology Criteria for Adverse Events (CTCAE) version 5.0, as determined by the treating physician.

^g^ Including grade 5 dermatitis combined with myositis/respiratory insufficiency/pancreatitis (*n* = 1).

Overall, 30.0% of patients (*n* = 61) experienced a grade 1–2 immune‐related adverse event (irAE), while for 19.2% of patients (*n* = 39), a grade ≥3 irAE was reported; the most common irAE was hepatitis (*n* = 8, 3.9%), followed by endocrine disorders and colitis (each *n* = 7, 3.4%). Five fatal events occurred; two of them (1 diabetic ketoacidosis and 1 enterocolitis) were reported as being associated with EV plus P (Table 3). To note, patients presenting with a high CCI prior to treatment initiation did not experience a higher frequency of SAEs in comparison to those with a low CCI (45.1 vs. 36.2%, *p* = .26).

In total, 108 patients (53.2%) had received EV at the full dose until discontinuation or data cut‐off date, while 93 patients (45.8%) required dose reductions to either 1 mg/kg up to 100 mg (*n* = 54, 26.6%), 0.75 mg/kg up to 75 mg (*n* = 29, 14.3%), or 0.5 mg/kg up to 50 mg (*n* = 10, 4.9%). The percentages of treatment discontinuation were quite similar for both therapies, mainly due to toxicities (EV: *n* = 39, 19.2%; P: *n* = 32, 15.8%) or progressive disease (EV: *n* = 35, 17.2%; P: *n* = 31, 15.3%). The remaining patients discontinued treatment at their own request or were lost to follow‐up (EV: *n* = 33, 16.2%; P: *n* = 26, 12.8%).

## 5 DISCUSSION

This first Austrian real‐world data confirm the high effectiveness and tolerability of EV plus P in routine clinical practice. After a median follow‐up period of approximately 2.5 years in the EV‐302 trial, the combination of EV plus P showed an ORR of 67.5% (95% CI, 62.9–71.9), including 30.4% CR and 37.1% PR.^12^ A DCR was achieved by 86.5% of the study population. The mPFS in the EV‐302 trial was 12.5 months (95% CI, 10.4–16.6) and the mOS was 33.8 months (95% CI, 26.1–39.3). In our real‐world cohort of Austrian patients, a comparable treatment response was observed; however, slightly lower DCR and CR rates were noted, while the PR rate was higher (63.6%), despite the relatively short follow‐up period. In the prespecified subgroup analyses of the EV‐302 trial, a decrease in mPFS and mOS was observed in patients presenting with less favourable ECOG PS 1–2 status compared to those with good ECOG PS 0.^13^ It is noteworthy that the treatment response was not affected by impaired kidney function, primary tumour histology, or the occurrence of SAEs.

In direct comparison to other retrospective real‐world analyses, for example, the retrospective real‐world UNITE US study, after a median follow‐up of 5.3 months, patients treated with frontline EV plus P showed a low ORR of 54% (95% CI, 41–65) but a higher DCR of 88%, compared with our population.^14^ Conversely, in another US real‐world retrospective study comprising a higher proportion of female patients with la/mUC, the ORR (76.0% in mUC, 81.8% in laUC) and the DCR (82.4% in mUC, 90.8% in laUC) were higher after 7.1 months of median follow‐up. In this study, the mPFS was 12.7 months (95% CI, 9.8‐NE) and the mOS was 25.1 months (95% CI, 16.3‐NE).^15^

In the German multicentre retrospective real‐world patient Guardians‐cohort, after a median follow‐up of 6.0 months, the ORR was 56.6% (with 10.3% CR and 46.3% PR) and the DCR 69.1%.^16^ These data, which were considerably lower than the outcomes observed in our analysis and in the EV‐302 trial, were somewhat unexpected, given that both studies dealt with a very similar population in terms of patient demographics and disease characteristics, except for the location of some metastases (lymph nodes, 74.6% vs. 36.9% in our analysis; liver, 22.4% vs. 18.7%, respectively). The mPFS was 13 months (95% CI, 5.2–20.8), while the mOS had not yet been reached.

With regard to safety, all‐grade skin disorders were observed in 37.4% of all patients analysed, which is lower than the rates previously reported in a systematic review and meta‐analysis of EV‐treated patients, which found that 49.7% of patients experienced skin toxicities.^17^ Although peripheral sensory neuropathy was the most common TRAEs observed in this analysis, it occurred at a lower frequency than reported in the EV‐302 trial (38.9 vs. 51.8%), but with a similar incidence to that observed in the Guardians cohort (42.3%).^12^, ^16^

Mayer et al. showed much higher discontinuation rates with EV (46.8 vs. 19.2%) and P (42.3 vs. 15.8%) compared to our data in a study of advanced UC patients treated with first‐line EV plus P in the United States. This outcome might be explained by the older age (median, 76 vs. 70 years) and less favorable ECOG PS (0–1, 76.3 vs. 84.2%; >2, 23.7 vs. 15.8%) of the analyzed US population.^18^

The present study is subject to certain limitations, the majority of which are inherent to its retrospective design. Furthermore, at the data cut‐off date, the median follow‐up period of less than 6 months precluded the assessment of mPFS and mOS because of the long‐term benefit of this combination therapy in this population.

Overall, the study findings reflect the significant changes in the treatment landscape for mUC as this novel combination has achieved the highest ORR and CR rates ever reported for this patient population. Ongoing trials addressing the non‐metastatic, muscle‐invasive setting with EV plus immune therapy will likely raise the CR rate benchmark in this setting and might help to generate solutions for bladder preservation strategies in the future.

## 6 CONCLUSIONS

This analysis shows that a high treatment response can also be achieved in a la/mUC population treated with EV plus P in daily routine. Even patients presenting with less favourable ECOG PS status, a high number of comorbidities, or impaired renal function could safely and effectively be treated. This is the major advantage compared to highly selected clinical study populations. It is therefore crucial to collect such real‐world data on the effectiveness and safety of this novel combination therapy to confirm the clinical trial findings in a large clinical routine setting.

Our real‐world data support the results of the pivotal study, with the need for extended follow‐up to estimate the mPFS/OS and the duration of response. Regarding response and treatment tolerability, the identification of prognostic and predictive biomarkers in future studies will be essential to optimise patient selection. Elucidating the pathophysiology of toxicities is paramount to prevent rare but sometimes fatal toxicities.

## AUTHOR CONTRIBUTIONS

**Dora Niedersuess‐Beke:** Conceptualization; methodology; data curation; investigation; validation; formal analysis; supervision; funding acquisition; visualization; project administration; writing – review and editing; writing – original draft; resources. **Karl Mayrhofer:** Methodology; software; data curation; investigation; validation; formal analysis; resources; writing – review and editing; visualization. **Johanna Krauter:** Data curation; investigation; resources; writing – review and editing. **Johannes Franke:** Data curation; investigation; resources; writing – review and editing. **Dominic Vais:** Data curation; investigation; resources; writing – review and editing. **Maximillian Pallauf:** Data curation; investigation; resources; writing – review and editing. **David Kiesl:** Data curation; investigation; resources; writing – review and editing. **Ferdinand Luger:** Data curation; investigation; resources; writing – review and editing. **Jacob Pfuner:** Data curation; investigation; resources; writing – review and editing. **Angelika Terbuch:** Data curation; investigation; resources; writing – review and editing. **Thomas Bauernhofer:** Data curation; investigation; resources; writing – review and editing. **Jasmin Spielgelberg:** Data curation; investigation; resources; writing – review and editing. **Andreas Banner:** Data curation; investigation; resources; writing – review and editing. **Stefan Aufderklamm:** Data curation; investigation; resources; writing – review and editing. **Clemens Wiesinger:** Data curation; investigation; resources; writing – review and editing. **Susanne Schnabel:** Data curation; investigation; resources; writing – review and editing. **Simon Peter Gampenrieder:** Data curation; investigation; resources; writing – review and editing. **Josef Mühlmann:** Data curation; investigation; resources; writing – review and editing. **Sonia Vallet:** Data curation; investigation; resources; writing – review and editing. **Sabine Weibrecht:** Data curation; investigation; resources; writing – review and editing. **Franz Stoiber:** Data curation; investigation; resources; writing – review and editing. **Haleh Andalibi:** Data curation; investigation; resources; writing – review and editing. **Harun Fajkovic:** Data curation; investigation; resources; writing – review and editing. **Hossein Taghizadeh:** Data curation; investigation; resources; writing – review and editing. **Jan Miechowiecki:** Data curation; investigation; resources; writing – review and editing. **Roman Taedcke:** Data curation; investigation; resources; writing – review and editing. **Daniel Heintel:** Investigation; resources; writing – review and editing; conceptualization; project administration. **Shahrokh F. Shariat:** Investigation; resources; writing – review and editing; conceptualization; validation; supervision; project administration. **Martin Pichler:** Investigation; resources; writing – review and editing; conceptualization. **Wolfgang Hilbe:** Data curation; investigation; resources; writing – review and editing. **Renate Pichler:** Data curation; investigation; resources; writing – review and editing; conceptualization; methodology; validation; supervision; visualization; project administration; writing – original draft.

## FUNDING INFORMATION

Astellas Pharma GmbH provided financial support for creating and maintaining the registry. ID: EV‐000019810.

## CONFLICT OF INTEREST STATEMENT

Dora Niedersuess‐Beke has received speakers and consultant honoraria from MSD and Astellas Pharma GmbH, as well as a research grant from Astellas Pharma GmbH. Johanna Krauter served as speaker and participated in advisory boards for Merck, and served as speaker for Astellas Pharma GmbH. Thomas Bauernhofer has received honoraria for lectures provided by Johnson & Johnson, AstraZeneca, Bayer, BMS, MSD, Novartis, Ipsen, Esai and Astellas Pharma GmbH. Andreas Banner has received honoraria from Astellas Pharma GmbH. Stefan Aufderklamm participated in advisory boards for Astellas Pharma GmbH and has received honoraria for lectures from Astellas Pharma GmbH. Clemens Wiesinger has received financial support for attending meetings from Bayer AG, Ferring GmbH, Janssen‐Cilag Pharma GmbH, AstraZeneca GmbH; he participated in advisory boards from Janssen‐Cilag Pharma GmbH. Simon Peter Gampenrieder received speaker honoraria from MSD, as well as consulting fees from Astellas Pharma GmbH and MSD. Maximillian Pallauf received honoraria for participating in advisory boards from BMS and AstraZeneca, as well as a travel grant from Johnson & Johnson and AstraZeneca. Josef Mühlmann received consultancy honoraria for Astellas Pharma GmbH. Sonia Vallet has received honoraria from Astellas Pharma GmbH and MSD. Shahrokh Shariat has received honoraria as speaker and consultant for Astellas Pharma GmbH and MSD. Harun Fajkovic has received research support from Johnson & Johnson, Bayer, Astellas Pharma GmbH, Takeda, Baxter and Arthrex; he has received consultancy fees from Johnson & Johnson, Astellas Pharma GmbH, IPSEN, MSD and AFS Medical; he was speaker for Johnson & Johnson, Bayer, Astellas Pharma GmbH, MSD and Boston Scientific; he has received honoraria from Johnson & Johnson, Astellas Pharma GmbH, IPSEN, Bayer, MSD and Takeda; and he participated in scientific advisory boards from Johnson & Johnson, Ferring, Astellas Pharma GmbH, IPSEN, Bayer and MSD. Renate Pichler participated in advisory boards from Astellas Pharma GmbH; she has received honoraria for lectures from Astellas Pharma GmbH and research grants from Astellas Pharma GmbH. The remaining authors have no relevant disclosures.

## ETHICS STATEMENT

All procedures were conducted in accordance with relevant local regulations and institutional guidelines. The patient registry approval from the central ethics committee of Vienna, Austria (EK‐22‐275‐VK) was initially granted on 28 June 2023, and then extended on 12 June 2024.

## Data Availability

The R code is publicly available on GitHub (https://github.com/KarlMayrhofer-MD/evpembro/blob/main/r-code). The other data that support the findings of this study are available from the corresponding author upon reasonable request.
